# LF3 exerts anti-osteosarcoma effects through Wnt signaling inhibition-coupled ferroptosis induction via *HO-1/ACSL4* axis

**DOI:** 10.3389/fonc.2026.1832247

**Published:** 2026-05-12

**Authors:** Mengfan Yang, Pei Liu, Hongjun Zhang, Xun Tang, Xiang Hong, Yujiao Liu, Meichao Deng, Xiaolin Tu, Gaohai Shao

**Affiliations:** 1 Department of Orthopedics, Affiliated Yongchuan Hospital of Chongqing Medical University, Chongqing, China; 2 Department of Obstetrics and Gynecology, Affiliated Yongchuan Hospital of Chongqing Medical University, Chongqing, China; 3 Key Laboratory of Diagnostic Medicine Designated by the Chinese Ministry of Education, College of Laboratory Medicine, Chongqing Medical University, Chongqing, China

**Keywords:** dual mechanism, ferroptosis, LF3, osteosarcoma, Wnt signaling

## Abstract

**Background:**

Osteosarcoma, the most common primary malignant bone tumor, predominantly affects adolescents and is characterized by high aggressiveness and early metastatic propensity. Chemoresistance and recurrence remain major clinical challenges, highlighting the urgent need for novel therapeutic targets and molecular mechanisms.

**Methods:**

Bioinformatics analysis of the GEO dataset (GSE42352) revealed a negative correlation between Wnt target gene expression and ferroptosis‑related gene signatures. The expression of Wnt and ferroptosis pathway genes/proteins, as well as autophagy and pyroptosis markers, was validated in 143B and MG63 cells by qRT‑PCR and Western blotting. ROS, Fe^2+^, and lipid peroxidation levels were measured using DCFH‑DA, FerroOrange, and Liperfluo, respectively. Mitochondrial morphology was assessed by transmission electron microscopy (TEM). Cell proliferation, migration, and invasion were evaluated by live/dead staining, wound healing assay, and Transwell invasion assay, respectively. Functional roles of HO‑1 and ACSL4 were validated via siRNA‑mediated knockdown in 143B cells. An osteosarcoma xenograft model was established; tumor expression of ACSL4, HO‑1, and Ki67 was examined by IHC, and serum ferritin levels were measured by ELISA.

**Results:**

Bioinformatics analysis of the GEO dataset (GSE42352) revealed a negative correlation between Wnt target gene expression and ferroptosis‑related gene signatures, suggesting that ferroptosis may be involved. In 143B and MG‑63 cells, LF3 (40 μM) downregulated Wnt target genes (AXIN2, BMP4, SMAD6, LEF1) and TCF4 protein expression, significantly inhibiting cell proliferation, migration, and invasion. LF3 treatment induced ROS elevation, lipid peroxidation, and characteristic mitochondrial morphological changes, independent of apoptosis, autophagy, or pyroptosis. Mechanistic studies demonstrated that LF3 activated ferroptosis through upregulation of HO‑1 and ACSL4/LPCAT3. In an osteosarcoma xenograft model, LF3 reduced tumor volume by approximately 60%, accompanied by elevated serum ferritin and tumor 4‑HNE levels; these effects were partially reversed by DFO co‑treatment. No systemic toxicity was observed.

**Conclusion:**

These findings establish LF3 as a dual-targeting agent that overcomes osteosarcoma through coordinated Wnt suppression and ferroptosis activation via the HO-1/ACSL4 axis, providing a novel therapeutic strategy for chemoresistant osteosarcoma.

## 1 Introduction

Osteosarcoma, the most common primary malignant bone tumor, predominantly affects adolescents and is characterized by high aggressiveness and early metastatic propensity (1). Approximately, 30% of patients present metastases at diagnosis, with a five-year survival rate below of 20% (2–4). Although surgical resection combined with neoadjuvant chemotherapy has improved survival rates, chemoresistance and recurrence remain major clinical challenges (5). Critically, the development of chemoresistance involves dysregulation of multiple signaling pathways that promote tumor survival and suppress cell death mechanisms. Thus, exploring novel therapeutic targets and molecular mechanisms, particularly those regulating chemoresistance and cell death pathways, is urgently needed.

The Wnt/β-catenin canonical pathway, a core regulator of cellular fate, governs osteosarcoma’s malignant behaviors through signaling cascades (6). Mechanistically, aberrant activation of this pathway disrupts β-catenin degradation complexes, reduces tumor cell adhesion, and promotes metastasis (7). Notably, its inhibition suppresses migration/invasion by downregulating MMPs (Matrix Metalloproteinases) activity and activates caspase cascades to induce programmed cell death (8). Existing evidence highlights the dysregulation of this signaling network as a critical molecular basis for osteosarcoma’s malignant phenotypes. Despite the established role of Wnt signaling in osteosarcoma, effective Wnt-targeting therapeutics remain limited in clinical application. Furthermore, emerging evidence suggests that Wnt pathway hyperactivation not only drives tumor progression but also modulates cellular iron metabolism and redox homeostasis—processes that are intimately connected to an alternative form of cell death that has recently attracted considerable attention.

This alternative mechanism is ferroptosis, an iron-dependent form of regulated cell death that has emerged as a promising strategy to overcome chemoresistance in cancer therapy (9–11). Morphologically, ferroptosis features shrunken mitochondria, increased membrane density, and reduced or absent cristae (12, 13). Unlike apoptosis, which is frequently inactivated in chemoresistant tumors, ferroptosis operates through a distinct iron- and lipid peroxidation-dependent mechanism that remains functional even in apoptosis-defective cells. Given that both Wnt signaling hyperactivation and ferroptosis resistance contribute to chemoresistance in osteosarcoma, we questioned whether these two pathways might be mechanistically interconnected. Studies have demonstrated that ferroptosis inducers Erastin and RSL3 significantly delay chemoresistant prostate cancer growth *in vivo (*14). Additionally, the PARP inhibitor Olaparib enhances therapeutic sensitivity in BRCA-wildtype ovarian cancer by downregulating SLC7A11 and synergizing with ferroptosis inducers to overcome chemoresistance (15). Multiple studies confirm that ferroptosis specifically targets therapy-resistant malignant cell populations (16), demonstrating therapeutic potential by modulating ferroptosis-related signaling to halt tumor progression. Given that both Wnt signaling hyperactivation and ferroptosis resistance contribute to chemoresistance in osteosarcoma, we questioned whether these two pathways might be mechanistically interconnected—and whether targeting Wnt signaling could provide a strategy to simultaneously suppress tumor progression and activate ferroptosis.

Emerging evidence suggests a previously underappreciated connection between Wnt signaling and ferroptosis regulation. The Wnt pathway modulates cellular iron homeostasis through multiple mechanisms: β-catenin activation enhances iron uptake and storage capacity, while Wnt target genes influence redox balance and lipid metabolism—processes central to ferroptosis execution (17). TCF/LEF transcription factors, the downstream effectors of canonical Wnt signaling, may also function as transcriptional regulators of ferroptosis-related genes, although this relationship has not been systematically investigated. In ovarian cancer, the Wnt receptor Frizzled-7 has been shown to promote ferroptosis by increasing intracellular labile iron (18). Conversely, Wnt/β-catenin activation confers ferroptosis resistance in gastric cancer through GPX4 upregulation (19). These context-dependent findings raise an intriguing possibility: in osteosarcoma, where Wnt signaling is constitutively activated, pharmacological Wnt inhibition might simultaneously suppress tumor progression and sensitize cells to ferroptosis—thereby addressing chemoresistance through a dual-targeting mechanism.

Based on these observations, we hypothesized that inhibiting Wnt signaling in osteosarcoma would not only suppress malignant behaviors but also activate ferroptosis as a secondary anti-tumor mechanism. To test this hypothesis, we selected LF3, a well-characterized small-molecule inhibitor that specifically disrupts the β-catenin/TCF4 interaction—the terminal transcriptional effector of canonical Wnt signaling (20). Unlike upstream Wnt inhibitors that target ligand secretion (Porcupine inhibitors) or receptor binding (anti-Frizzled antibodies), LF3 acts at the transcriptional level, directly blocking Wnt-dependent gene expression regardless of the mechanism of pathway activation (21). This property is particularly relevant for osteosarcoma, where Wnt hyperactivation can occur through multiple mechanisms including receptor overexpression, ligand autocrine signaling, and mutations in destruction complex components (22). Furthermore, LF3 has demonstrated favorable efficacy and tolerability in preclinical cancer models, supporting its translational potential. This study aimed to: (1) confirm LF3’s inhibitory effects on Wnt signaling and malignant phenotypes in osteosarcoma; (2) determine whether LF3 induces ferroptosis; and (3) elucidate the molecular mechanism connecting Wnt inhibition to ferroptosis activation.

## 2 Materials and methods

### 2.1 Reagent

The following chemicals were purchased from MedChemExpress (NJ, USA): LF3 (Cat. No. HY-19956), deferoxamine (DFO, Cat. No. HY-B0988), ferrostatin-1 (Fer-1, Cat. No. HY-100579), Q-VD-OPH (Cat. No. HY-12305), and Z-VAD-FMK (Cat. No. HY-16658). Calcein/PI Live/Dead Viability/Cytotoxicity Assay Kit was purchased from Beyotime Biotechnology (Shanghai, China). CCK-8 Assay Kit was purchased from MedChemExpress (NJ, USA). FerroOrange, DCFH-DA ROS Assay Kit, and Liperfluo were obtained from Dojindo (Beijing, China). Serum ferritin ELISA Kit was purchased from Mengbio (Chongqing, China).

### 2.2 Cell culture

Human osteosarcoma cell lines 143B and MG-63 were purchased from iCell Bioscience Inc. (Shanghai, China) and cultured in DMEM (Gibco, NY, USA) supplemented with 10% fetal bovine serum (FBS; Wisent, Montreal, Canada) and 1% penicillin-streptomycin (Beyotime Biotechnology, Shanghai, China) at 37 °C in a 5% CO_2_ incubator.

### 2.3 Cell viability assay

Cell viability was assessed using the CCK-8 assay per the manufacturer’s protocol²³. Briefly, cells were seeded in 96-well plates and incubated with CCK-8 reagent for 30 min at 37 °C. Absorbance was measured at 450 nm using a Varioskan LUX microplate reader (Thermo Fisher Scientific, USA).

### 2.4 Calcein/PI live/dead viability/cytotoxicity assay

Cells were stained with 100 μL staining solution per well and incubated at 37 °C for 30 min in the dark. Fluorescence was measured using a Varioskan LUX microplate reader (Thermo Fisher Scientific, USA): calcein (live cells) at Ex/Em 494/517 nm (FITC channel) and propidium iodide (PI; dead cells) at Ex/Em 535/617 nm (TRITC channel).

### 2.5 Detection of cell apoptosis

Apoptosis was assessed by Annexin V-FITC/PI double staining using a CytoFlex flow cytometer. Cells were collected, washed with PBS, stained with Annexin V-FITC and PI for 15 min in the dark, and analyzed by flow cytometry.

### 2.6 Real time quantitative polymerase chain reaction

Total RNA was extracted using TRIzol reagent (Accurate Biology, Hunan, China) and reverse-transcribed using the Evo M-MLV Reverse Transcription Premix Kit (Accurate Biology). cDNA was diluted 5-fold and stored at −20 °C until use. RT-qPCR was performed using the SYBR Green Premium Pro Taq HS RT-qPCR Kit on a CFX Connect system (Bio-Rad, USA). Primer sequences are provided in **Supplementary Table 1**. mRNA expression was normalized to GAPDH using the 2^−ΔΔCT^ method (23).

### 2.7 Western blot

Total protein was extracted using RIPA lysis buffer (Beyotime Biotechnology) supplemented with 1% PMSF and 1% phosphatase inhibitor. Thirty micrograms of protein per sample were resolved on 10% SDS-PAGE gels (Epizyme Biotech, Shanghai, China) and transferred to 0.2 μm PVDF membranes (Boster Biological Technology, Wuhan, China). Membranes were blocked with 5% BSA for 90 min at room temperature, then incubated with primary antibodies overnight at 4 °C: anti-HO-1 (Cat. No. ab13243, Abcam, 1:1000), anti-ACSL4 (Cat. No. ab155282, Abcam, 1:1000), anti-TCF4 (Cat. No. 2569, CST, 1:1000), and anti-β-actin (Cat. No. 4970, CST, 1:5000). After incubation with HRP-conjugated goat anti-rabbit secondary antibody (90 min, room temperature), signals were detected using ECL reagent (Abbkine Scientific, Wuhan, China) on a ChemiScope S6 imaging system (CLINX, Shanghai, China). Band intensities were quantified using ImageJ.

### 2.8 Analyses of high-throughput sequencing data

Transcriptomic data from the GSE42352 dataset (106 osteosarcoma samples, 12 normal bone samples) were downloaded from the NCBI GEO database. Differentially expressed genes (DEGs) were identified using the limma package in R (v4.2.0) with thresholds of log_2_ FC≥1.0 and FDR < 0.05. Samples were stratified into Wnt high- and low-expression groups based on the expression patterns of key Wnt target genes. Ferroptosis-related gene signatures were curated from the FerrDb V2 database (http://www.zhounan.org/ferrdb). Venn diagram analysis identified overlapping genes between Wnt-associated DEGs and ferroptosis regulators. Data visualization was performed using the ggplot2 package in R, with Log2(x+1) normalization applied for inter-group comparability. Pearson correlation analysis assessed the relationship between Wnt target gene expression and ferroptosis gene signatures.

### 2.9 Detection of intracellular Fe^2+^ fluorescence

The treated cells were added into 1 μmol/L detection solution (300 μL/well) and incubated at 37°C for about 30 minutes (24). Observations were made directly under an inverted fluorescence microscope without other processing, and images were captured and saved. Image J software was used for the quantification of analysis.

### 2.10 Detection of intracellular reactive oxygen species

Culture medium was removed and cells were washed twice. Remove the supernatant and add the prepared working solution. Cells were incubated for 30 minutes at 37 °C under 5% CO_2_ conditions. Remove the working solution, wash the cells twice again and perform detection by inverted fluorescence microscope (25). Image J software was used for the quantification of analysis.

### 2.11 Detection of lipid peroxidation

The measurement of lipid peroxidation was conducted using Liperfluo, which is a modified version of a lipid hydrogen peroxide probe (26, 27). The procedure was as follows (28): After the cells were rinsed with PBS 1x buffer for 3 times, a 2 μM Liperfluo working solution diluted in PBS 1x buffer was applied and incubated for 30 minutes at 37 °C. Images were captured using a fluorescence microscope or lipid peroxidation was quantified using flow cytometry. Image J software was used for the quantification of analysis.

### 2.12 Histology and immunohistochemistry

Paraffin-embedded tissue sections (5 μm) were baked at 62 °C, deparaffinized in xylene, and rehydrated through a graded ethanol series. For H&E staining, standard protocols were followed. For immunohistochemistry (IHC), microwave-based antigen retrieval was performed, followed by blocking of endogenous peroxidase activity with 3% hydrogen peroxide and non-specific binding with 10% BSA (30 min). Sections were incubated with primary antibodies (**Supplementary Table 3**) overnight at 4 °C, then with secondary antibodies for 50 min at 37 °C. Visualization was achieved with DAB chromogen solution (Zhongshan Jinqiao, Shanghai, China), and nuclei were counterstained with hematoxylin.

### 2.13 Cellular migration assay

Cells were seeded in plates and cultured until a confluent monolayer formed. A standardized scratch wound was created using a sterile 100 μL pipette tip. The monolayer was gently washed with 1x PBS to remove detached cells and debris, followed by the addition of serum-free or low-serum medium to minimize cell proliferation effects. Baseline images were captured immediately (0 h) using phase-contrast microscopy under consistent conditions. Sequential imaging was performed at 12 h, 24 h, and 48 h intervals, ensuring standardized positioning parameters throughout the experiment (29). Image J software was used for the quantification of analysis.

### 2.14 Transwell invasion assay

The upper chamber (8 μm pore size) was coated with Matrigel (1:8 dilution in serum-free DMEM) and polymerized at 37 °C for 3 h. Serum-starved cells were resuspended in serum-free medium, seeded into the upper chamber, and drug-treated DMEM was added to the lower chamber. After 48 h, non-invaded cells were removed with a cotton swab. Invaded cells on the lower membrane surface were fixed with 4% paraformaldehyde, stained with crystal violet, and imaged under a microscope³¹. Invaded cell numbers were quantified using ImageJ.

### 2.15 Transmission electron microscopy assays

143B and MG-63 cells were trypsinized, centrifuged at 225 × g for 5 min, and fixed with 4% glutaraldehyde at 4 °C for 2 h, followed by 1% osmium tetroxide fixation at 4 °C for 1 h. Cells were dehydrated through graded ethanol and acetone series and embedded in Epon 816 (Electron Microscopy Sciences, Hatfield, PA, USA). Ultrathin sections were cut using a Leica ultramicrotome (Leica Microsystems, Buffalo Grove, IL, USA), stained with uranyl acetate and lead citrate, and examined under a JEM-1400Plus transmission electron microscope (JEOL Ltd., Tokyo, Japan)³².

### 2.16 siRNA transfection

siRNA duplexes were synthesized by GenaPharma (Shanghai, China). Sequences were as follows: ACSL4-siRNA sense 5′-GCAGAGAUAUCUUGCUUUATT-3′, antisense 5′-UAAAGCAAGAUAUCUCUGCTT-3′; HO-1-siRNA sense 5′-GGAGAUUGAGCGCAACAAGTT-3′, antisense 5′-CUUGUUGCGCUCAAUCUCCTT-3′; NC-siRNA sense 5′-UCCUCCGAACGUGUCACGUTT-3′, antisense 5′-ACGUGACACGUUCGGAGAATT-3′. 143B cells were seeded in six-well plates at 1 × 10^5^ cells/well. At 60% confluence, cells were transfected with siRNA (100 nM final concentration) using Lipofectamine™ 2000 (Invitrogen, USA) per the manufacturer’s instructions. After 8 h, medium was replaced with DMEM containing 10% FBS. Cells were harvested 48 h post-transfection for subsequent experiments³³. All transfections were performed in three independent biological replicates.

### 2.17 Animal study

The animal study protocol was approved by the Institutional Animal Care and Use of Chongqing Medical University (Approval Number: IACUC-CQMU-2024-0346). All experimental procedures were performed in accordance with the Institutional Guide for the Care and Use of Laboratory Animals. Twenty 4-week-old athymic nude mice were procured from Changzhou Cavens Experimental Animal Co., Ltd. (Jiangsu, China) and maintained in a specified pathogen-free (SPF) facility. Following acclimation under a 12-h light/dark cycle with ad libitum access to food and water for one week, 5×10^6^ 143B cells suspended in 100 μL PBS were subcutaneously inoculated into the mice. After one week, the mice were randomly allocated into three groups (n=6 mice per group): control group received intraperitoneal injections of equivalent volume saline, the LF3 group received LF3 (50 mg/kg), and the DFO group received combined LF3 (50 mg/kg) and DFO (10 mg/kg) (19, 20). Treatments were administered daily for three weeks. Subsequently, euthanized mice underwent tumor tissue resection and serum collection for further analysis.

### 2.18 Ethics statement and animal procedures

All animal experiments were conducted in accordance with the Guide for the Care and Use of Laboratory Animals and approved by the IACUC of Chongqing Medical University (Approval No. IACUC-CQMU-2024-0346). Mice requiring sedation were anesthetized with 2% isoflurane in 100% oxygen at 1 L/min. At study completion, animals were euthanized by CO_2_ inhalation (30–70% chamber volume/min) followed by cervical dislocation as secondary confirmation.

### 2.19 ELISA assay

Peripheral blood was incubated overnight at 4 °C and centrifuged (7,000 rpm, 15 min, 4 °C) to isolate serum. Standards were prepared immediately before use. Each well received 10 μL sample or standard plus 40 μL dilution buffer, followed by 100 μL HRP-conjugated detection antibody and incubation at 37 °C for 60 min. After five washes, 50 μL each of substrates A and B were added for chromogenic development (37 °C, 15 min, dark). The reaction was stopped with 50 μL stop solution, and OD450 was measured within 15 min.

### 2.20 Statistical analysis

Statistical analyses were performed using GraphPad Prism (version 9.0, GraphPad Software, San Diego, CA, USA). Data are presented as mean ± standard deviation (SD). For comparisons between two groups, unpaired two-tailed Student’s t-test was applied. For comparisons among three or more groups, one-way analysis of variance (ANOVA) followed by Tukey’s *post-hoc* test was used. For tumor growth curve analysis, two-way ANOVA with Bonferroni correction was applied. Bioinformatics correlation analyses were performed using Pearson correlation coefficient. A P value < 0.05 was considered statistically significant. *P < 0.05, **P < 0.01, ***P < 0.001.

## 3 Results

### 3.1 Bioinformatics analysis reveals a potential regulatory link between Wnt signaling and ferroptosis in osteosarcoma

To establish the rationale for investigating Wnt-ferroptosis crosstalk, we performed bioinformatics analysis using the GSE42352 dataset. We hypothesized that if Wnt signaling regulates ferroptosis sensitivity, Wnt pathway activity should inversely correlate with ferroptosis-related gene expression in osteosarcoma.

**Figure 1:**
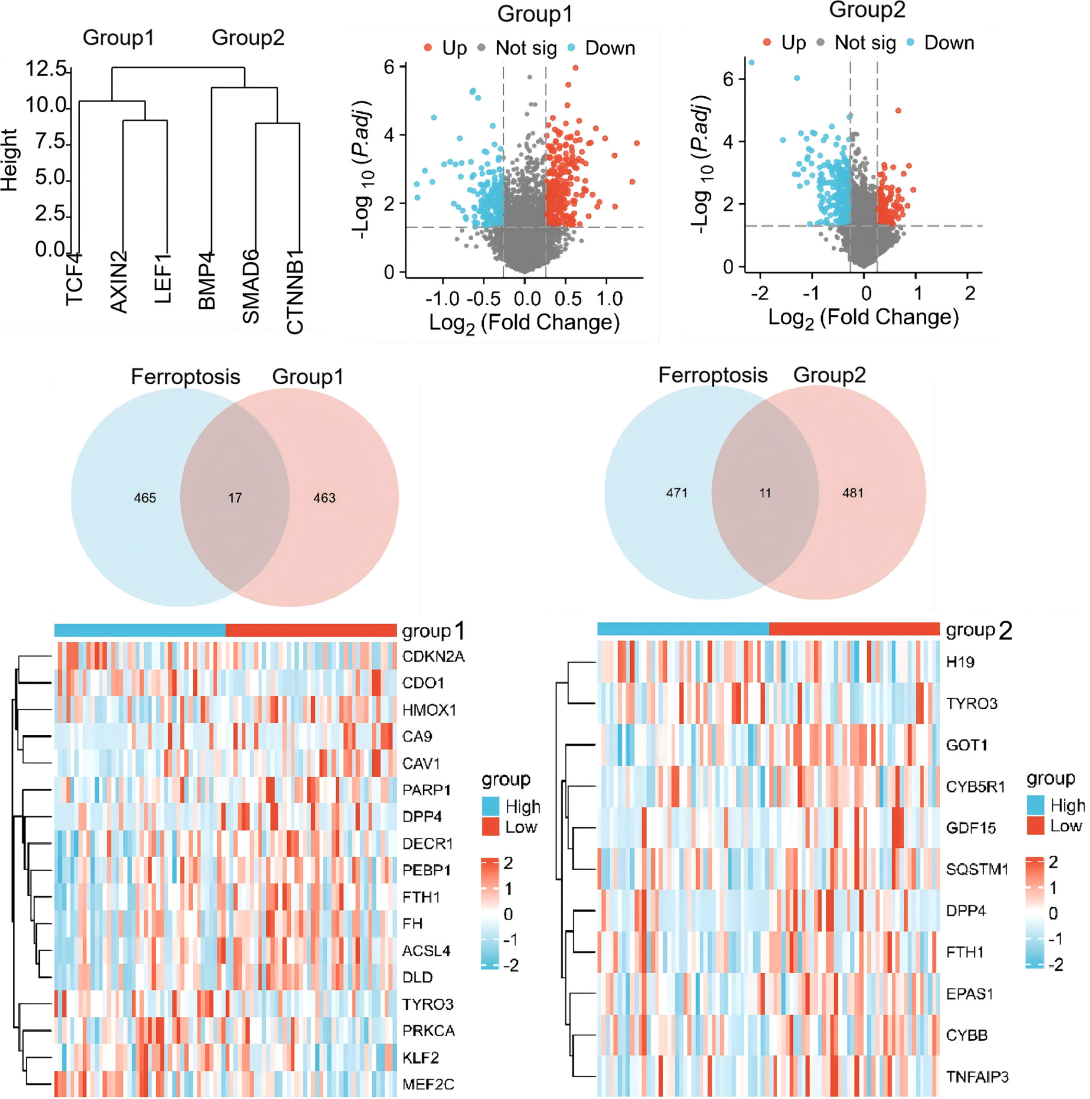
Bioinformatics analysis reveals crosstalk between Wnt signaling and ferroptosis in osteosarcoma. To investigate the potential relationship between Wnt signaling and ferroptosis in osteosarcoma, transcriptomic data from the GEO dataset GSE42352 were analyzed. Hierarchical clustering analysis was performed on Wnt target genes in osteosarcoma versus normal bone tissue samples. Differentially expressed genes (DEGs) were identified using the thresholds of |log_2_ fold change| ≥ 1.0 and adjusted P value (FDR) < 0.05, and subsequently cross-referenced with the ferroptosis-related gene database (FerrDb V2). Venn diagram analysis illustrates the overlap between Wnt-associated DEGs and ferroptosis-related genes. Heatmap analysis visualizes the expression patterns of overlapping genes, revealing a previously unrecognized transcriptional association between the Wnt signaling pathway and ferroptosis in osteosarcoma.

Hierarchical clustering of Wnt signaling components identified two functional gene modules: Group 1 (TCF4, AXIN2, LEF1) and Group 2 (BMP4, SMAD6, CTNNB1). Samples were stratified into Wnt high- and low-expression groups, followed by intersection analysis with ferroptosis regulators from the FerrDb database. This revealed significant overlap: 17 ferroptosis-related genes were associated with Group 1 and 11 with Group 2 (**Figure 1**). Overlapping genes included HO-1, a key enzyme in heme degradation and iron release, and FTH1, the primary intracellular iron storage protein—suggesting that Wnt activity may inversely correlate with ferroptosis susceptibility in osteosarcoma.

### 3.2 LF3 effectively suppresses Wnt/β-catenin signaling in osteosarcoma cells

**Figure 2:**
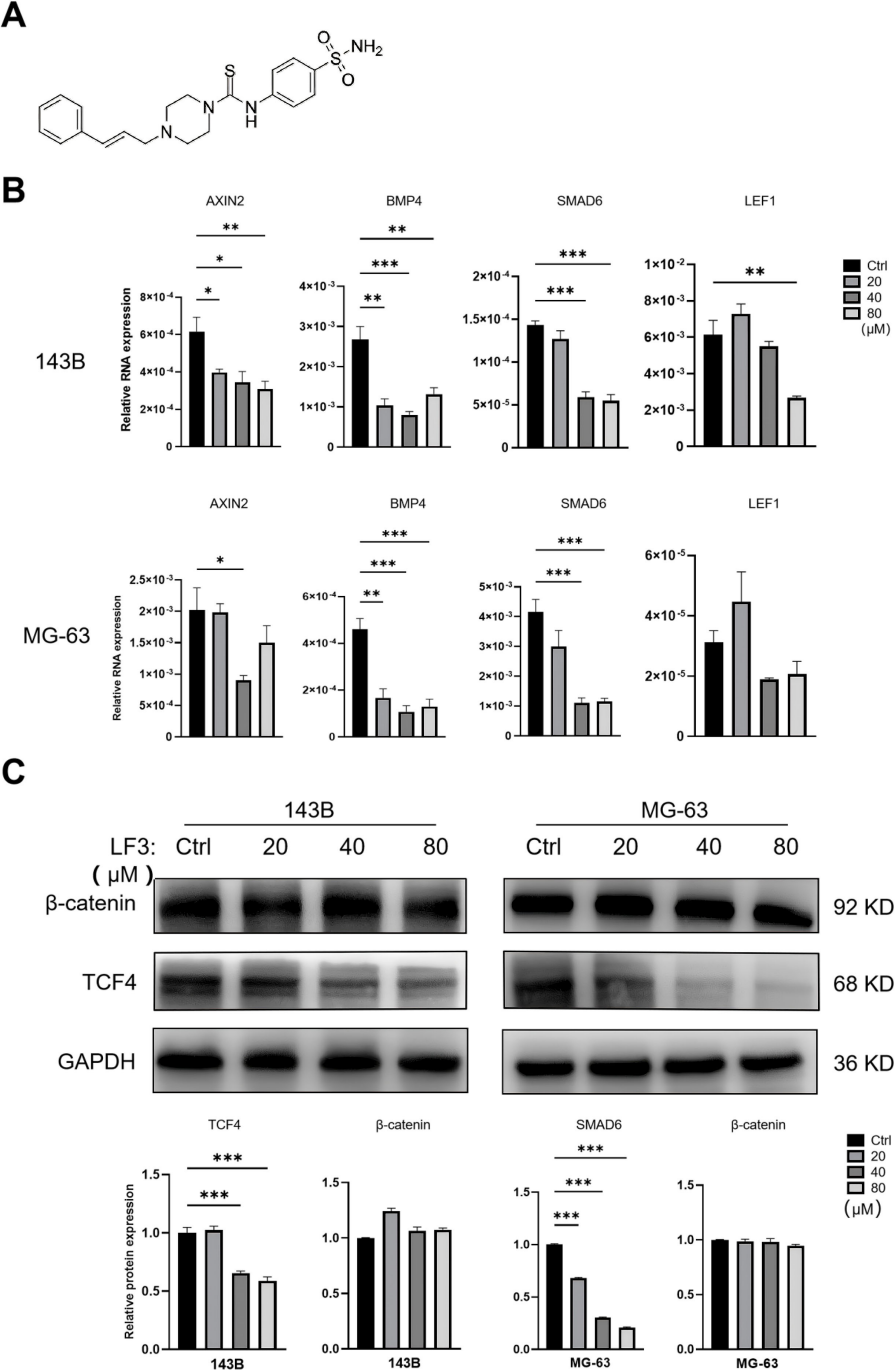
LF3 inhibits the Wnt/β-catenin signaling pathway via the β-catenin/TCF4 axis. To evaluate the effect of LF3 on Wnt/β-catenin signaling, 143B and MG-63 osteosarcoma cells were treated with increasing concentrations of LF3 (20, 40, 80 μM) or DMSO (vehicle control) for 48 h, followed by qPCR and Western blot (WB) analyses. **(A)** Chemical structure of LF3, a small-molecule antagonist of the β-catenin/TCF4 protein-protein interaction. **(B)** qPCR analysis demonstrating LF3-mediated dose-dependent transcriptional suppression of Wnt target genes AXIN2, BMP4, SMAD6, and LEF1 in both 143B and MG-63 cells. **(C)** Western blot analysis revealing dose-dependent downregulation of TCF4 protein expression following LF3 treatment, while total β-catenin protein levels remain unaltered, indicating that LF3 disrupts the β-catenin/TCF4 interaction without affecting β-catenin protein stability. Representative blot images and corresponding densitometric quantification are shown. Data are presented as mean ± SD. *P < 0.05, **P < 0.01, ***P < 0.001 vs. control group; n = 3 independent experiments.

Having identified a potential Wnt-ferroptosis connection, we confirmed that LF3 effectively inhibits Wnt signaling in osteosarcoma cells. LF3 (**Figure 2A**) disrupts the β-catenin/TCF4 protein-protein interaction, blocking Wnt-dependent transcription without affecting upstream components. Both 143B and MG-63 cell lines were selected to represent different metastatic potentials and genetic backgrounds.

Treatment with LF3 (20, 40, 80 μM, 48 h) dose-dependently downregulated Wnt target genes AXIN2, BMP4, SMAD6, and LEF1 in both cell lines, with significant differences at 40 μM (**Figure 2B**). Western blot further demonstrated dose-dependent reduction of TCF4 protein expression, while β-catenin levels remained unchanged (**Figure 2C**). Together, these results confirm that LF3 suppresses Wnt signaling through TCF4 reduction rather than β-catenin degradation.

**Figure 3:**
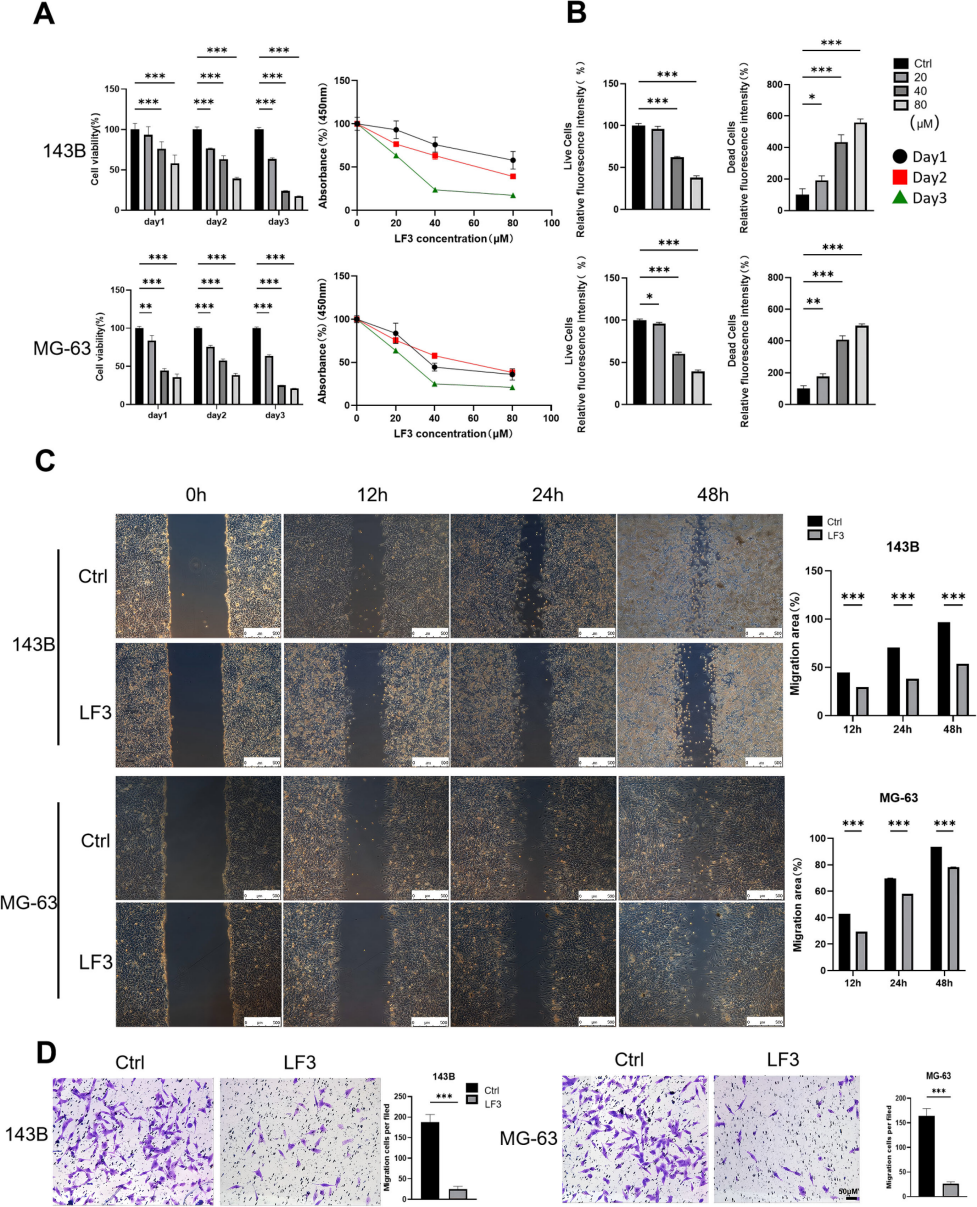
LF3 inhibits the proliferation, migration, and invasion of osteosarcoma cells. To evaluate the anti-tumor effects of LF3 on osteosarcoma malignant phenotypes, 143B and MG-63 cells were treated with LF3 at indicated concentrations for 48 h. **(A)** CCK-8 assay demonstrating concentration-dependent inhibition of cell viability in 143B and MG-63 cells treated with LF3 (20, 40, 80 μM) for 48 h. IC_50_ values were calculated using nonlinear regression analysis. **(B)** Live/dead cell staining showing a significant reduction in viable cell proportion (calcein AM-positive, green) and a corresponding increase in dead cell proportion (PI-positive, red) following LF3 treatment (40 μM, 48 h) in both cell lines. Scale bar = 100 μm. **(C)** Wound healing assay revealing significantly suppressed cell migration distance in LF3-treated cells (40 μM, 48 h) compared to DMSO-treated controls, with representative images taken at 0 h and 24 h after wound creation. Scale bar = 200 μm. **(D)** Transwell invasion assay indicating significantly reduced invasive cell numbers in LF3-treated cells (40 μM, 48 h) compared to controls. Representative images of crystal violet-stained invaded cells are shown. Scale bar = 100 μm. Data are presented as mean ± SD. *P < 0.05, **P < 0.01, ***P < 0.001 vs. control group; n = 3 independent experiments.

Based on CCK-8 dose-response curves, estimated IC values for LF3 at 48 h treatment were approximately 55-65 μM for 143B cells and 50-60 μM for MG-63 cells. 40 μM was the closest experimental concentration to IC at which cell viability was reduced to approximately 50-60%, resulting in biologically significant and statistically robust effects while avoiding possible non-specific toxicity at high concentrations of 80 μM, and was therefore chosen for subsequent mechanistic experiments (**Figure 3A**).

### 3.3 Wnt inhibition by LF3 induces ferroptosis in osteosarcoma cells

To test whether LF3-mediated Wnt inhibition triggers ferroptosis, we examined ferroptosis-related gene expression and functional hallmarks in LF3-treated osteosarcoma cells.

**Figure 4:**
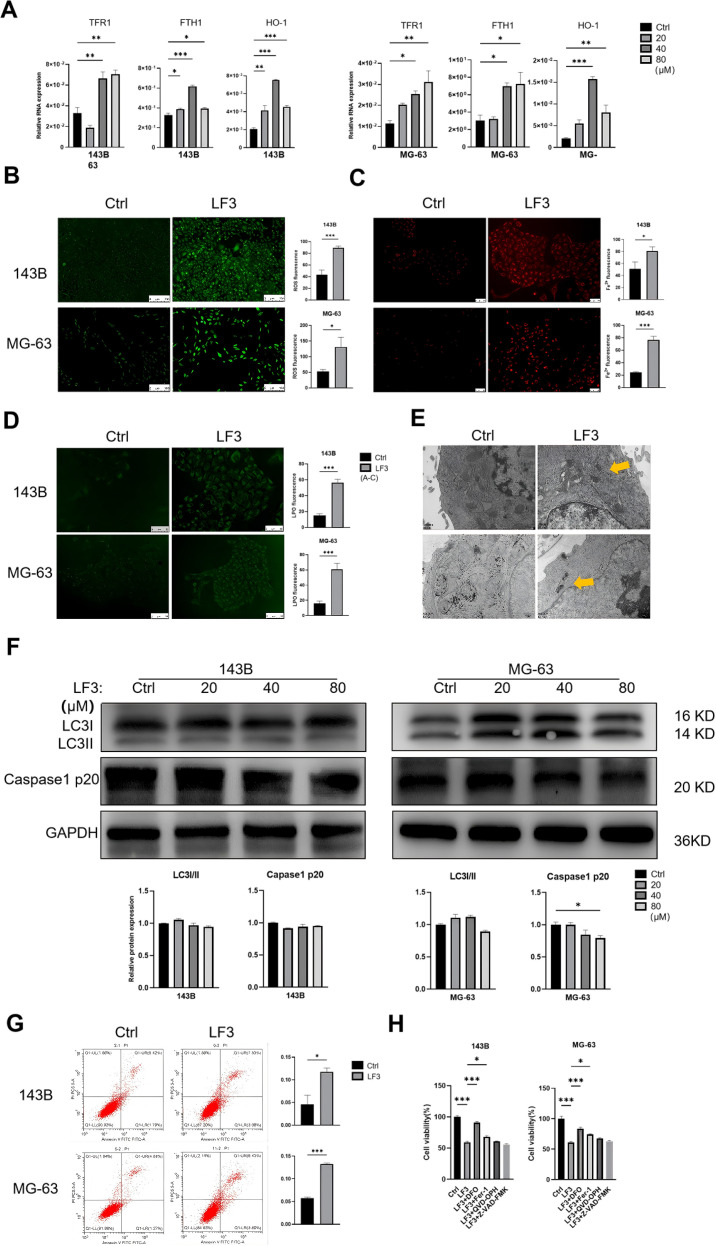
Wnt inhibition by LF3 induces ferroptosis in osteosarcoma cells. **(A)** qPCR analysis of TFR 1, HO-1, and FTH 1 expression levels in LF3-treated osteosarcoma cells. **(B)** Intracellular ROS accumulation in 143B and MG-63 cells were detected using the fluorescent probe DCFH-DA (scale bar = 100 μm). **(C)** Intracellular Fe^2+^ accumulation in 143B and MG-63 cells were measured by the fluorescent probe FerroOrange (scale bar = 100 μm). **(D)** Lipid peroxidation in 143B and MG-63 cells were assessed using the fluorescent probe Liperfluo (scale bar = 50 μm). **(E)** Transmission electron microscopy (TEM) revealed that LF3-treated cells exhibited mitochondrial damage, including loss or fragmentation of cristae, compared to control groups. The arrow indicates mitochondria exhibiting ferroptotic features. **(F)** Western blot showed no significant changes in autophagy-related protein LC3-I/II or pyroptosis marker Caspase1 p20. **(G)** Flow cytometry with Annexin V/PI staining indicated a low proportion of apoptotic cells. **(H)** CCK-8 assay demonstrated that co-treatment with apoptosis inhibitors (Q-VD-OPH 20 μM, Z-VAD-FMK 20 μM) failed to rescue the proliferative capacity of LF3-treated osteosarcoma cells. Data are presented as mean ± SD. *P < 0.05, **P < 0.01, ***P < 0.001; n = 3 independent experiments.

qPCR analysis of 143B and MG-63 cells treated with LF3 (20, 40, 80 μM, 48 h) revealed dose-dependent upregulation of *TFR1, HO-1*, and *FTH1* at 40 μM (**Figure 4A**), consistent with ferroptosis activation. Functionally, LF3 (40 μM, 48 h) significantly elevated intracellular ROS (DCFH-DA staining, **Figure 4B**), labile iron accumulation (FerroOrange staining, **Figure 4C**), and lipid peroxidation (Liperfluo staining, **Figure 4D**). TEM further confirmed characteristic ferroptotic mitochondrial changes, including increased membrane density and cristae loss (**Figure 4E**).

To exclude other cell death mechanisms, Western blot showed no significant changes in autophagy marker LC3A/B or pyroptosis marker Caspase-1 p20 (**Figure 4F**). Flow cytometry revealed that cumulative apoptotic cell proportions remained below 15% following 40 μM LF3 treatment (30, 31) (**Figure 4G**). Rescue experiments confirmed that apoptosis inhibitors Q-VD-OPH and Z-VAD-FMK failed to restore cell viability, whereas ferroptosis inhibitor Fer-1 provided partial rescue (**Figure 4H**). Collectively, these results demonstrate that LF3 induces ferroptosis as the primary cell death mechanism in osteosarcoma cells.

### 3.4 LF3 suppresses malignant phenotypes of osteosarcoma cells through ferroptosis-dependent and -independent mechanisms

LF3 treatment (20, 40, 80 μM, 48 h) dose-dependently reduced cell viability in both 143B and MG-63 cells, with significant effects at 40 μM by CCK-8 assay (**Figure 3A**). Live/dead staining confirmed a marked reduction in viable cell proportion with a corresponding increase in dead cells (**Figure 3B**). Wound healing and Transwell invasion assays demonstrated that 40 μM LF3 significantly suppressed cell migration and invasion compared to controls (**Figures 3C, D**).

**Figure 5:**
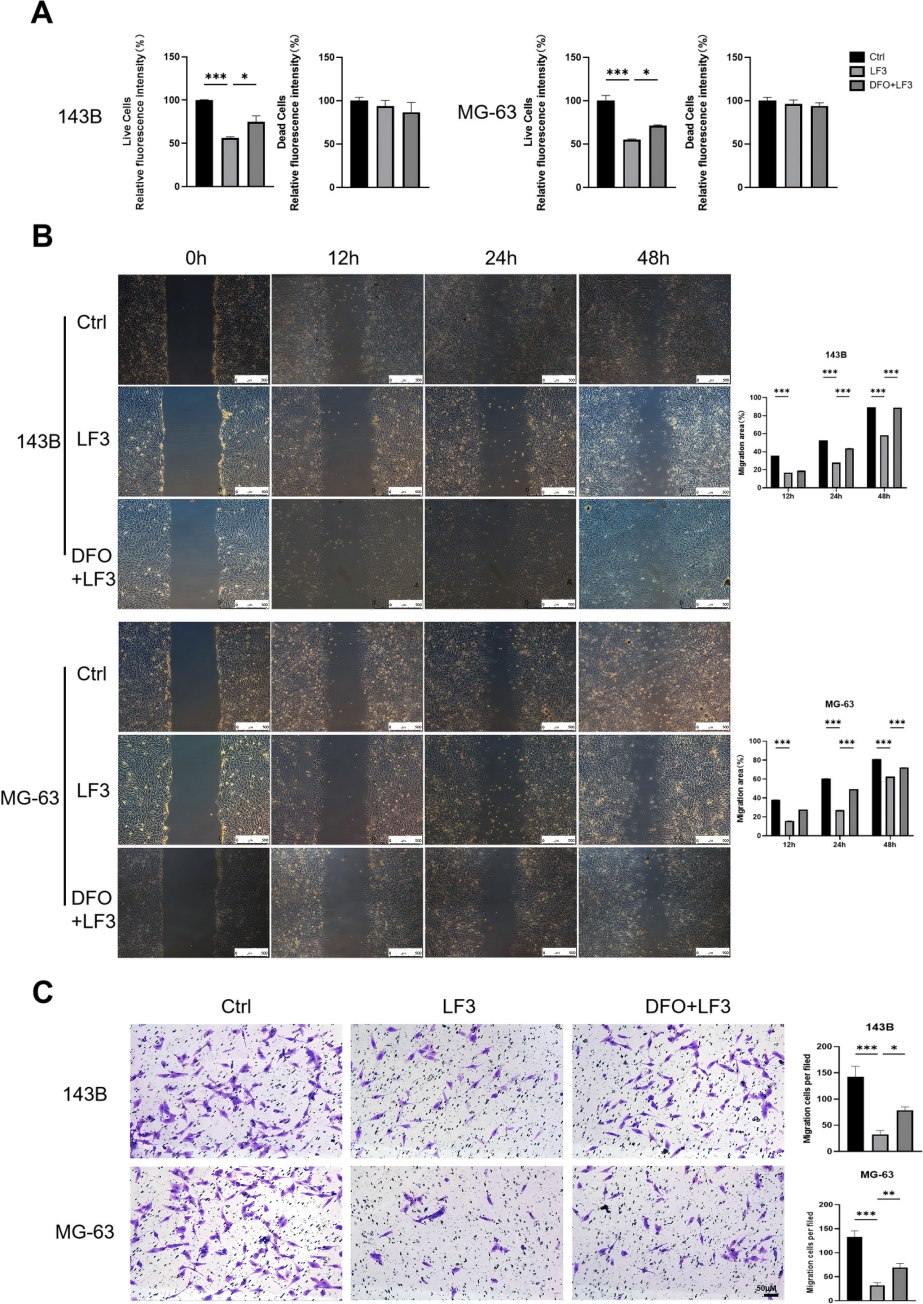
DFO rescues osteosarcoma cell viability. To determine the contribution of ferroptosis to LF3-induced inhibition of malignant phenotypes, 143B and MG-63 cells were treated with 40 μM LF3 alone (LF3 group), 40 μM LF3 combined with 20 μM deferoxamine (DFO, an iron chelator and ferroptosis inhibitor; LF3 + DFO group), or an equivalent volume of DMSO (vehicle control group) for 48 h, followed by assessments of cell viability, migration, and invasion. **(A)** Live/dead cell staining demonstrating that DFO co-treatment significantly increased the proportion of viable cells (calcein AM-positive, green) compared to LF3 treatment alone, confirming that ferroptosis contributes substantially to LF3-induced cytotoxicity. Scale bar = 100 μm. **(B)** Wound healing assay revealing partial restoration of migratory capacity in the LF3 + DFO group compared to the LF3 group. Representative images taken at 0 h and 24 h after wound creation are shown. Scale bar = 200 μm. **(C)** Transwell invasion assay demonstrating partial recovery of invasive ability in the LF3 + DFO group compared to the LF3 group. Representative images of crystal violet-stained invaded cells are shown. Scale bar = 100 μm. Data are presented as mean ± SD. *P < 0.05, **P < 0.01, ***P < 0.001; n = 3 independent experiments.

To dissect the contribution of ferroptosis to these anti-tumor effects, DFO co-treatment (20 μM, 48 h) significantly restored cell viability (**Figure 5A**), migration (**Figure 5B**), and invasion capacity (**Figure 5C**) compared to LF3 alone. These results demonstrate that LF3 suppresses osteosarcoma malignant phenotypes through combined Wnt-inhibitory and ferroptosis-inducing mechanisms.

### 3.5 DFO-mediated ferroptosis reversal confirms iron-dependent cell death mechanism

**Figure 6:**
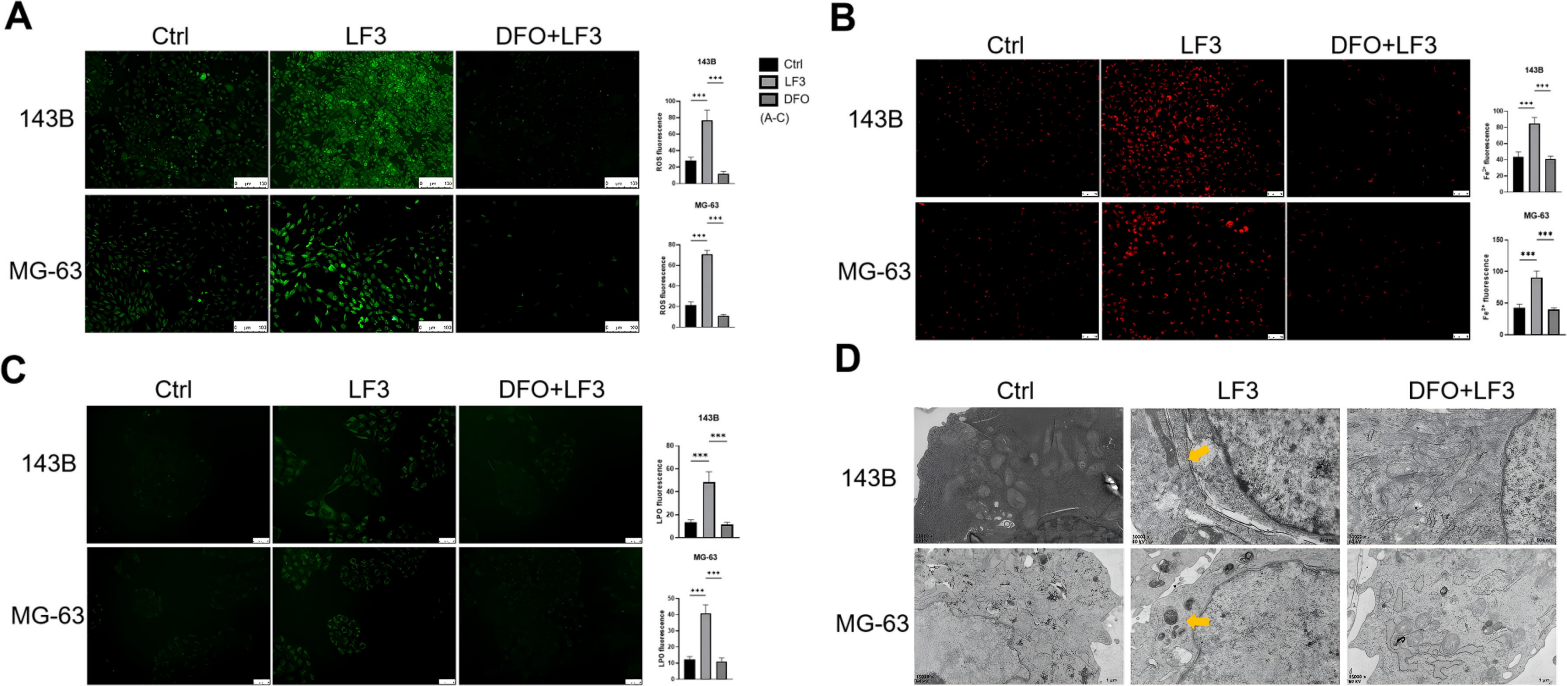
DFO antagonizes LF3-induced ferroptosis in osteosarcoma cells. To confirm that DFO specifically reverses LF3-induced ferroptosis through iron chelation, 143B and MG-63 cells were treated with 40 μM LF3 alone (LF3 group), 40 μM LF3 combined with 20 μM DFO (LF3 + DFO group), or DMSO (vehicle control group) for 48 h, followed by comprehensive ferroptosis marker analyses. **(A)** Intracellular ROS levels detected by DCFH-DA fluorescent probe, showing that DFO co-treatment significantly attenuated LF3-induced ROS accumulation in both 143B and MG-63 cells. Representative fluorescence microscopy images are shown. Scale bar = 100 μm. Fluorescence intensity was quantified using ImageJ and normalized to the control group. **(B)** Intracellular Fe^2+^ levels measured by FerroOrange fluorescent staining, demonstrating that DFO co-treatment markedly reduced LF3-induced intracellular iron accumulation. Representative fluorescence microscopy images are shown. Scale bar = 100 μm. Fluorescence intensity was quantified using ImageJ and normalized to the control group. **(C)** Lipid peroxidation levels assessed by Liperfluo fluorescent probe, showing that DFO co-treatment significantly suppressed LF3-induced lipid peroxidation in both cell lines. Representative fluorescence microscopy images are shown. Scale bar = 50 μm. Fluorescence intensity was quantified using ImageJ and normalized to the control group. **(D)** TEM images revealing that DFO co-treatment partially reversed LF3-induced ferroptotic mitochondrial morphological alterations, with preservation of mitochondrial membrane integrity and cristae structure compared to the LF3 group. Arrows indicate mitochondria exhibiting ferroptotic ultrastructural features. Data are presented as mean ± SD. ***P < 0.001; n = 3 independent experiments.

To confirm iron dependence of LF3-induced ferroptosis, we examined whether DFO co-treatment reverses molecular ferroptosis markers. DFO significantly reduced LF3-induced intracellular Fe^2+^ accumulation (**Figure 6A**), ROS elevation (**Figure 6B**), and lipid peroxidation (**Figure 6C**) in both cell lines. TEM confirmed that DFO preserved normal mitochondrial morphology with intact membranes and cristae, in contrast to the ferroptotic ultrastructural damage observed in LF3-treated cells (**Figure 6D**). These results establish that LF3-induced ferroptosis is strictly iron-dependent.

### 3.6 LF3 induces ferroptosis through coordinated regulation of *HO-1* and *ACSL4/LPCAT3*

**Figure 7:**
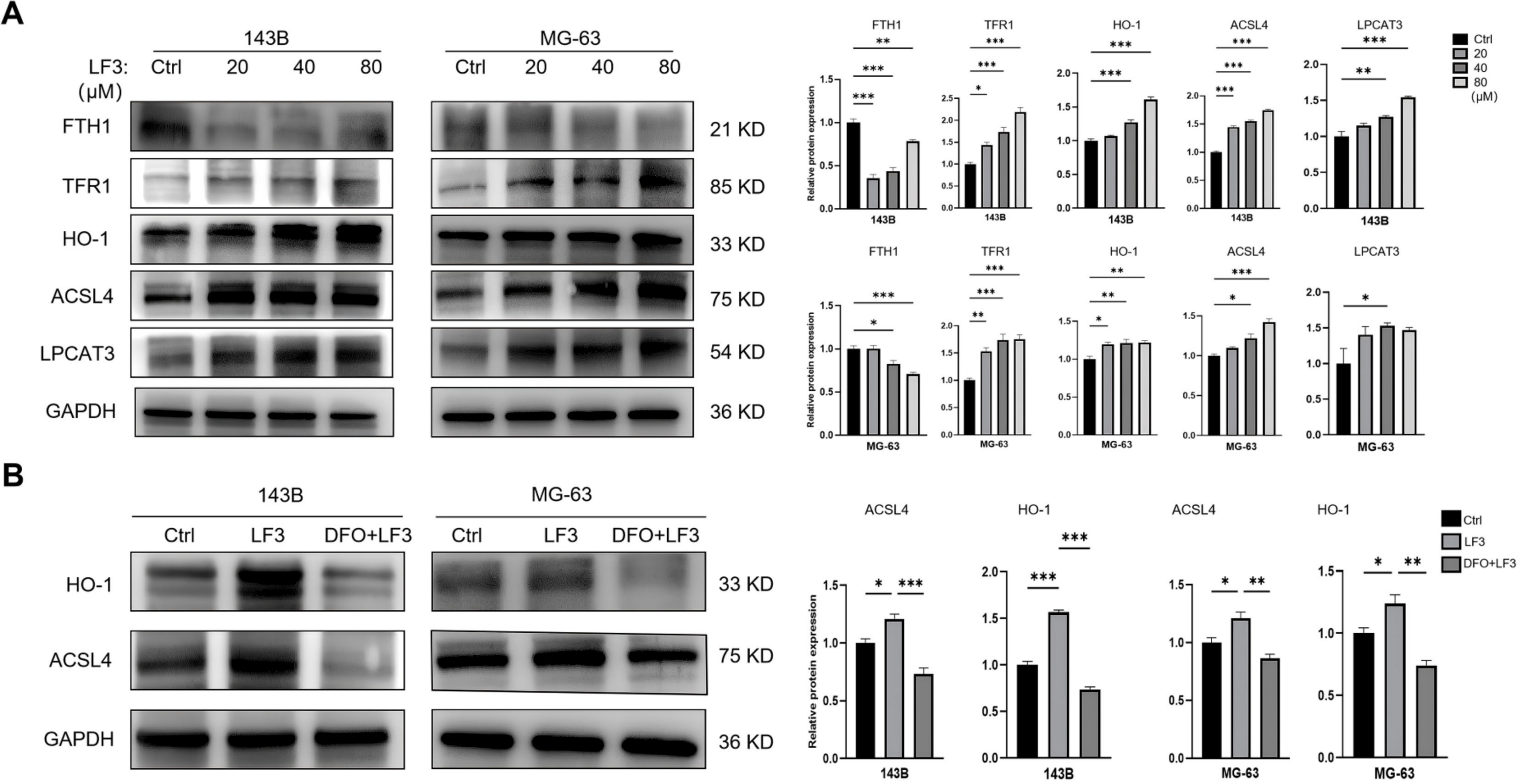
LF3 induces osteosarcoma ferroptosis via the HO-1 and ACSL4. To elucidate the molecular mechanism underlying LF3-induced ferroptosis, key ferroptosis-related proteins were examined by Western blot analysis. **(A)** Western blot analysis of ferroptosis-related proteins in 143B and MG-63 cells treated with increasing concentrations of LF3 (20, 40, 80 μM) or DMSO for 48 h. LF3 dose-dependently upregulated pro-ferroptotic proteins TFR1 (iron uptake), HO-1 (heme degradation and iron release), ACSL4, and LPCAT3 (lipid peroxidation), while downregulating the anti-ferroptotic iron storage protein FTH1. Representative blot images and corresponding densitometric quantification are shown. β-actin was used as a loading control. **(B)** Western blot analysis showing that co-treatment with 20 μM DFO significantly reversed LF3 (40 μM, 48 h)-induced upregulation of HO-1 and ACSL4 protein expression, suggesting these proteins act as downstream effectors in the iron-dependent ferroptotic cascade. Representative blot images and corresponding densitometric quantification are shown. Data are presented as mean ± SD. *P < 0.05, **P < 0.01, ***P < 0.001; n = 3 independent experiments.

Western blot analysis of LF3-treated cells (20, 40, 80 μM, 48 h) revealed dose-dependent upregulation of pro-ferroptotic proteins TFR1, HO-1, ACSL4, and LPCAT3, alongside downregulation of anti-ferroptotic FTH1 (**Figure 7A**). DFO co-treatment (20 μM) significantly reversed LF3-induced upregulation of HO-1 and ACSL4 (**Figure 7B**), identifying these proteins as downstream effectors in the ferroptotic cascade.

**Figure 8:**
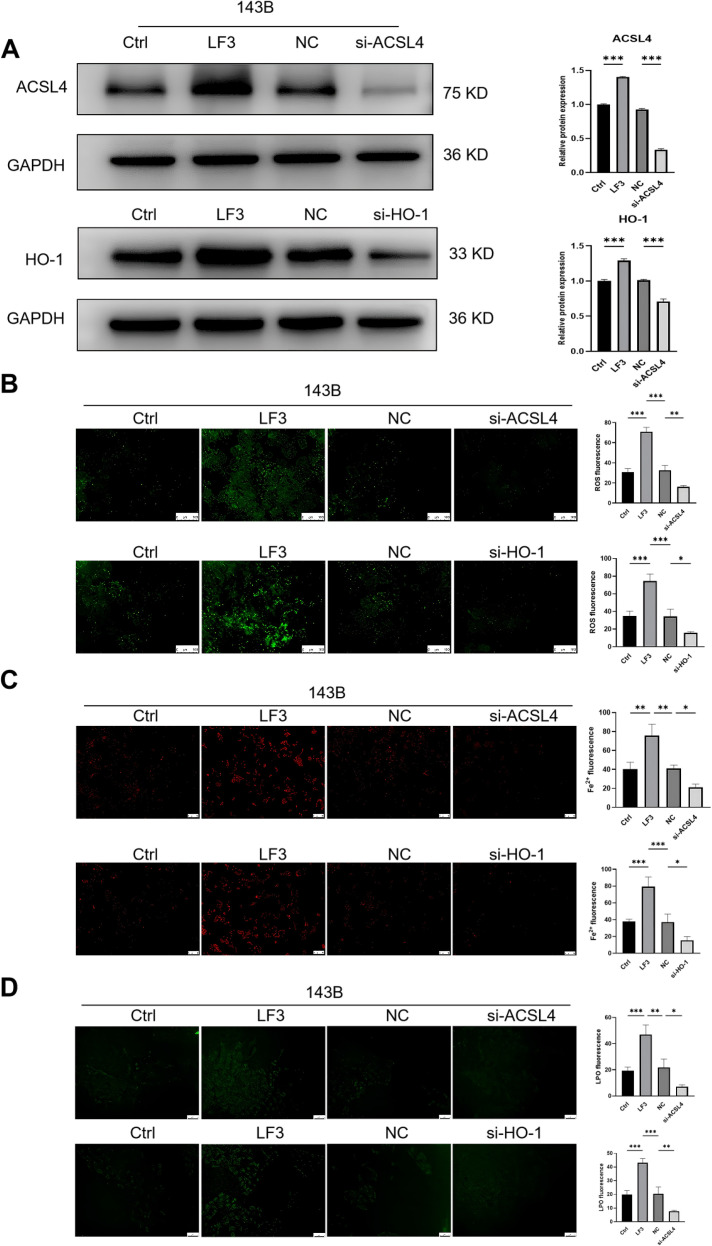
Knockdown of HO-1 and ACSL4 inhibits LF3-induced ferroptosis in osteosarcoma cells. To directly validate the functional roles of HO-1 and ACSL4 in LF3-induced ferroptosis, siRNA-mediated knockdown was performed in 143B cells prior to LF3 treatment (40 μM, 48 h). **(A)** Western blot confirming successful knockdown of HO-1 and ACSL4 protein expression following siRNA transfection compared to scrambled siRNA (negative control). β-actin was used as a loading control. **(B)** Intracellular ROS accumulation detected by DCFH-DA fluorescent probe in siRNA-transfected cells following LF3 treatment. Knockdown of HO-1 or ACSL4 significantly attenuated LF3-induced ROS elevation compared to the siRNA negative control group. Representative fluorescence microscopy images are shown. Scale bar = 100 μm. Fluorescence intensity was quantified using ImageJ and normalized to the control group. **(C)** Intracellular Fe^2+^ accumulation measured by FerroOrange fluorescent staining, demonstrating that HO-1 or ACSL4 knockdown significantly reduced LF3-induced intracellular iron accumulation. Representative fluorescence microscopy images are shown. Scale bar = 100 μm. Fluorescence intensity was quantified using ImageJ and normalized to the control group. **(D)** Lipid peroxidation levels assessed by Liperfluo fluorescent probe, showing that HO-1 or ACSL4 knockdown significantly suppressed LF3-induced lipid peroxidation. Representative fluorescence microscopy images are shown. Scale bar = 50 μm. Fluorescence intensity was quantified using ImageJ and normalized to the control group. Data are presented as mean ± SD. *P < 0.05, **P < 0.01, ***P < 0.001 vs. siRNA negative control + LF3 group; n = 3 independent experiments.

siRNA-mediated knockdown of HO-1 or ACSL4 in 143B cells (**Figure 8A**) significantly attenuated LF3-induced ROS accumulation (**Figure 8B**), Fe^2+^ elevation (**Figure 8C**), and lipid peroxidation (**Figure 8D**). These results establish that LF3 activates ferroptosis through two coordinated mechanisms: HO-1-mediated heme degradation expands the labile iron pool, while ACSL4/LPCAT3-mediated PUFA incorporation into membrane phospholipids provides substrates for lipid peroxidation.

### 3.7 *In vivo* validation confirms LF3 as a ferroptosis-inducing therapeutic agent for osteosarcoma

**Figure 9:**
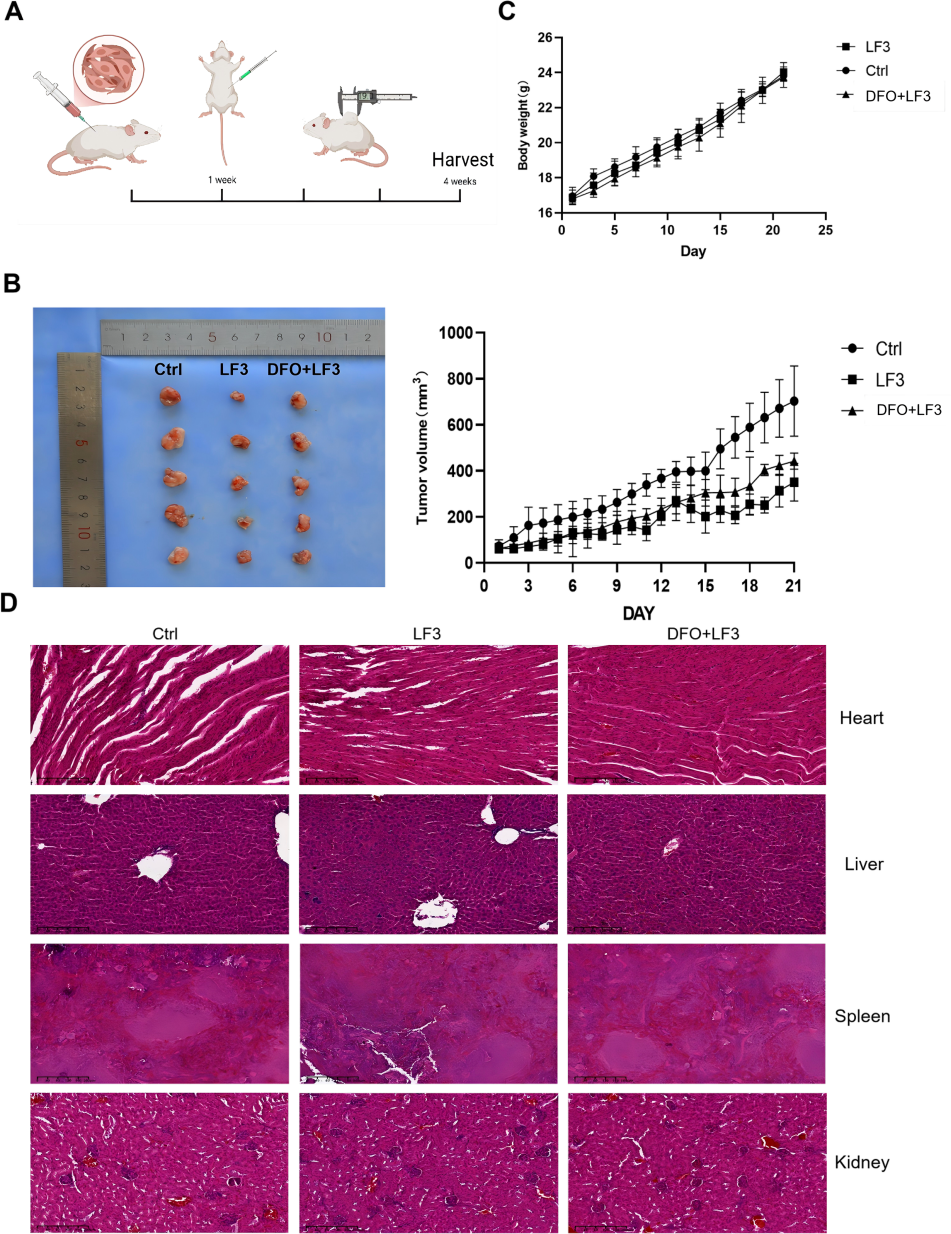
LF3 induces ferroptosis in osteosarcoma xenograft models *in vivo*. To evaluate the anti-tumor efficacy and ferroptosis-inducing activity of LF3 *in vivo*, a subcutaneous xenograft mouse model was established by injecting 143B cells into nude mice. Once tumors reached approximately 100 mm³, mice were randomly allocated into three groups: control (DMSO, i.p.), LF3 (20 mg/kg, i.p., every other day), and LF3 + DFO (LF3–20 mg/kg + DFO 100 mg/kg, i.p., every other day) for 28 days. **(A)** Schematic diagram illustrating the xenograft experimental design, including cell inoculation, grouping, drug administration schedule, and endpoint assessments. **(B)** Tumor growth curves showing tumor volume (mm³) measured every three days over the 28-day treatment period, and representative photographs of excised tumors at the experimental endpoint. LF3 treatment significantly reduced tumor volume compared to the control group, and DFO co-treatment partially reversed this inhibitory effect. **(C)** Body weight monitoring over the treatment period, demonstrating no significant treatment-related body weight loss, indicating acceptable systemic tolerability of LF3. **(D)** Hematoxylin and eosin (H&E) staining of major organs including heart, liver, spleen, lung, and kidney harvested at the experimental endpoint, showing no significant pathological damage or organ toxicity in any treatment group. Scale bar = 100 μm. Data are presented as mean ± SD. *P < 0.05, **P < 0.01, ***P < 0.001; n = 6 mice per group.

A subcutaneous 143B xenograft model was established in nude mice. Seven days post-inoculation, mice were randomized into three groups (n = 6/group) receiving vehicle, LF3, or LF3 + DFO for three weeks (**Figure 9A**). LF3 treatment reduced tumor volume by approximately 60% compared to vehicle control, and DFO co-administration partially reversed this suppression, confirming ferroptosis-dependent anti-tumor activity *in vivo* (**Figure 9B**). No mortality, significant body weight changes (**Figure 9C**), or organ pathology (**Figure 9D**) were observed, indicating a favorable safety profile.

**Figure 10:**
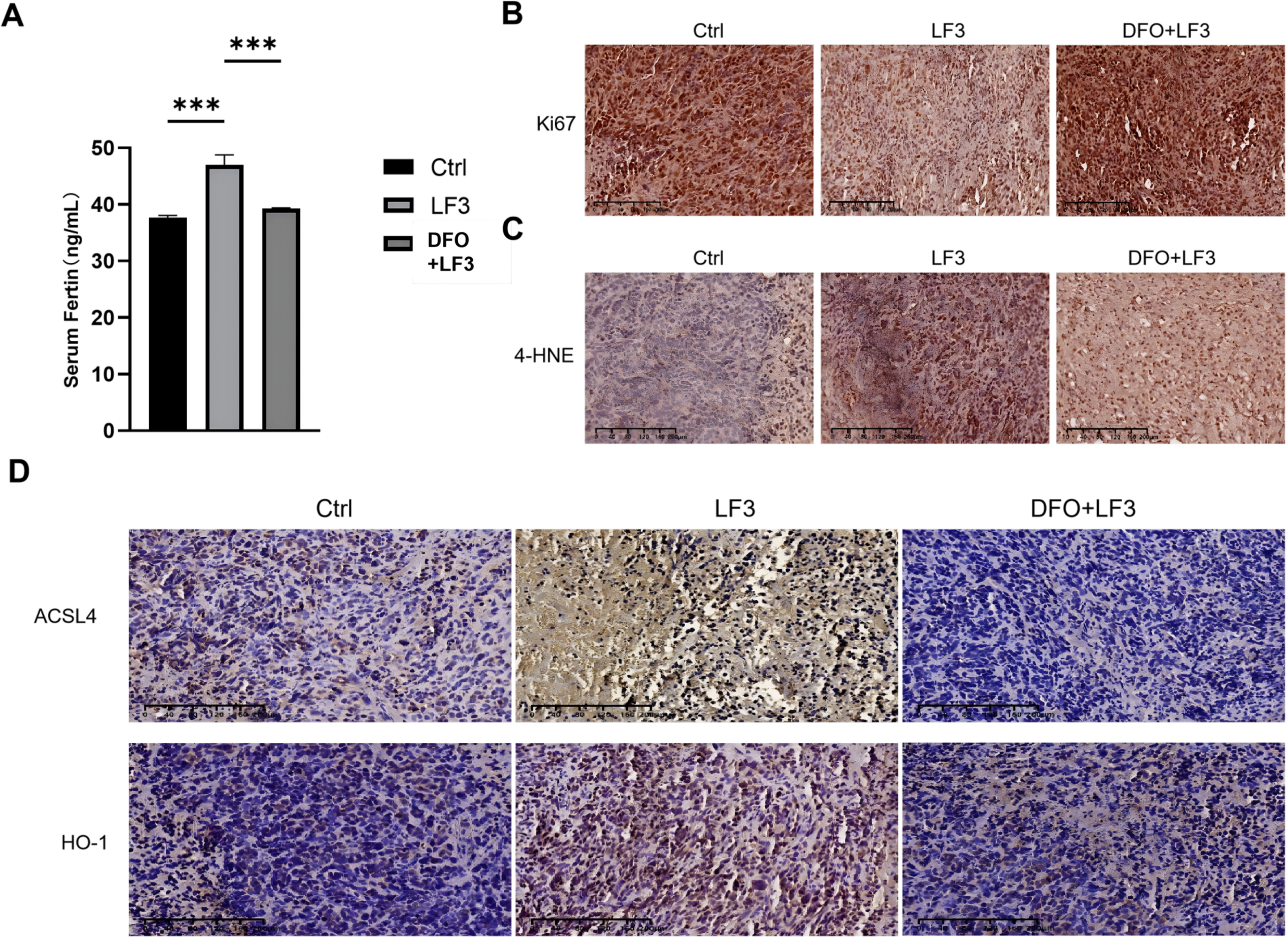
Systemic toxicity evaluation and *in vivo* mechanistic validation of LF3 and DFO. To validate the ferroptosis mechanism identified *in vitro* in the *in vivo* setting, tumor tissues and serum samples were collected from xenograft mice at the experimental endpoint and subjected to biochemical and immunohistochemical analyses. **(A)** Serum ferritin levels quantified by enzyme-linked immunosorbent assay (ELISA), demonstrating significantly elevated serum ferritin in LF3-treated mice compared to the control group, indicative of systemic iron mobilization consistent with ferroptosis activation. DFO co-treatment partially reversed this elevation. **(B)** Immunohistochemical (IHC) staining of tumor sections for Ki67, a proliferation marker, demonstrating a significant reduction in Ki67-positive cells in LF3-treated tumors compared to controls, confirming the anti-proliferative effect of LF3 *in vivo*. DFO co-treatment partially restored Ki67 positivity. Representative IHC images and corresponding quantification of positive cell percentage are shown. Scale bar = 100 μm. **(C)** IHC staining for 4-hydroxynonenal (4-HNE), a lipid peroxidation end-product and established ferroptosis biomarker, showing significantly upregulated 4-HNE expression in LF3-treated tumor tissues compared to controls. DFO co-treatment attenuated this upregulation, confirming ferroptosis as the underlying mechanism. Representative IHC images and corresponding quantification of staining intensity are shown. Scale bar = 100 μm. **(D)** IHC staining of tumor sections for ACSL4 and HO-1, the key mechanistic effectors identified *in vitro*, demonstrating that LF3 treatment significantly upregulated ACSL4 and HO-1 protein expression in osteosarcoma tissues *in vivo*, while DFO co-treatment suppressed this upregulation, corroborating the *in vitro* mechanistic findings and confirming the functional relevance of the HO-1/ACSL4 axis in LF3-induced ferroptosis *in vivo*. Representative IHC images and corresponding quantification of staining intensity (H-score) are shown. Scale bar = 100 μm. Data are presented as mean ± SD. *P < 0.05, **P < 0.01, ***P < 0.001; n = 6 mice per group.

Mechanistic analyses confirmed that LF3 elevated serum ferritin levels (**Figure 10A**), reduced Ki67-positive proliferating cells (**Figure 10B**), and increased tumor 4-HNE accumulation (**Figure 10C**)—all consistent with ferroptosis activation *in vivo*. IHC further demonstrated upregulation of HO-1 and ACSL4 in LF3-treated tumors, which was attenuated by DFO co-treatment (**Figure 10D**), corroborating the *in vitro* HO-1/ACSL4 ferroptosis mechanism. Collectively, these findings support LF3 as a promising ferroptosis-inducing therapeutic agent for osteosarcoma.

## 4 Discussion and conclusion

This study demonstrates that LF3, a small-molecule Wnt signaling inhibitor, exerts potent anti-osteosarcoma effects through a dual mechanism involving Wnt pathway suppression and ferroptosis induction via the *HO-1/ACSL4* axis. This dual-targeting approach provides a promising strategy for overcoming chemoresistance in osteosarcoma treatment.

LF3 effectively suppressed the canonical Wnt/β-catenin pathway in osteosarcoma cells, as evidenced by significant downregulation of Wnt target genes *AXIN2, BMP4, SMAD6*, and *LEF1*. By disrupting the *β-catenin/TCF4* interaction, LF3 blocks Wnt-dependent transcription without affecting β-catenin protein stability-distinguishing it from upstream Wnt inhibitors and enabling targeted suppression at the transcriptional effector level (20). Aberrant Wnt activation is well-established in osteosarcoma pathogenesis and correlates with poor prognosis (32, 33), supporting Wnt inhibition as a rational therapeutic strategy. Notably, bioinformatics analysis of clinical osteosarcoma samples revealed an inverse correlation between Wnt target gene expression and ferroptosis-related gene signatures, suggesting a previously uncharacterized regulatory crosstalk warranting further investigation.

The most significant finding of this study is that Wnt inhibition by LF3 triggers ferroptosis through coordinated upregulation of *HO-1* and the *ACSL4/LPCAT3* axis. LF3 induced hallmark ferroptotic features-iron accumulation, ROS generation, lipid peroxidation, and characteristic mitochondrial ultrastructural changes (9):independently of apoptosis, autophagy, and pyroptosis, as confirmed by inhibitor rescue assays and absence of LC3-I/II or Caspase-1 p20 alterations. The specificity of this ferroptotic mechanism was validated by DFO rescue experiments, which reversed both ferroptotic markers and anti-tumor effects, and by siRNA-mediated knockdown of *HO-1* and *ACSL4*, which significantly attenuated ROS accumulation, iron overload, and lipid peroxidation.

Mechanistically, LF3 upregulates HO-1, releasing free iron from heme catabolism to expand the labile iron pool, while simultaneously downregulating the iron storage protein FTH1-collectively elevating intracellular Fe^2+^ levels (34, 35). Concurrent upregulation of *ACSL4* and *LPCAT3* promotes incorporation of polyunsaturated fatty acids (PUFAs) into membrane phospholipids, sensitizing cells to lipid peroxidation (36, 37). This dual convergence of iron dysregulation and lipid remodeling creates a synergistic ferroptotic environment. Based on the available data, it cannot be judged whether the upregulation of HO-1 and ACSL4 stems from direct transcriptional de-repression of the TCF4 binding site or is an indirect effect mediated via Nrf2/ARE activation or Wnt-dependent lipid metabolism reprogramming. Computational analysis of promoter regions identified putative TCF/LEF binding motifs in HMOX1 and ACSL4 regulatory regions, supporting the feasibility of direct transcriptional regulation; however, experimental validation by ChIP-seq and promoter reporter assays is still required.

How *Wnt/TCF4* inhibition leads to *HO-1* and *ACSL4/LPCAT3* upregulation remains to be fully elucidated. Three plausible mechanisms are proposed: (1) TCF4 may directly repress ferroptosis gene promoters via β-catenin/TCF4-Groucho/TLE co-repressor complexes (38), such that LF3-mediated disruption relieves this repression; (2) Wnt inhibition may activate compensatory *Nrf2/ARE* signaling, upregulating *HO-1*—though paradoxically, excessive HO-1 activity promotes ferroptosis through iron release (34, 35, 39); (3) Wnt suppression may reprogram lipid metabolism via *SREBP* and *PPARγ* pathways, increasing ACSL4-mediated PUFA incorporation (36, 40). These hypotheses warrant experimental validation through ChIP-seq analysis of TCF4 binding sites and transcriptomic profiling of LF3-treated cells.

The Wnt-ferroptosis connection represents an emerging paradigm in cancer biology. While *Wnt/β-catenin* activation has been shown to confer ferroptosis resistance through GPX4 upregulation in certain cancers (19), our findings demonstrate the converse in osteosarcoma—Wnt inhibition sensitizes cells to ferroptosis through the HO-1/ACSL4 axis. This tissue-specific divergence likely reflects differences in downstream effector expression and baseline iron homeostasis across cancer types (41).

The selection of LF3 over upstream Wnt inhibitors offers three strategic advantages for osteosarcoma therapy: (1) pathway specificity, selectively blocking canonical Wnt signaling without affecting non-canonical branches (42); (2) bypass of upstream resistance, circumventing alternative receptor activation or ligand-independent β-catenin stabilization; and (3) dual functionality, simultaneously inhibiting Wnt-dependent transcription and activating ferroptosis—a property not previously reported for other Wnt inhibitors, distinguishing LF3 from agents such as ICG-001.

*In vivo* xenograft studies validated the translational potential of LF3, demonstrating approximately 60% tumor volume reduction with minimal systemic toxicity—no mortality, body weight loss, or organ pathology was observed. Elevated serum ferritin, tumor *4-HNE, HO-1*, and *ACSL4* levels confirmed that the *HO-1/ACSL4* ferroptosis mechanism remains operative *in vivo*. Partial reversal by DFO co-treatment indicates that Wnt inhibition independently contributes to tumor suppression beyond ferroptosis induction, supporting the dual mechanism model (43).

Current osteosarcoma treatment relies heavily on conventional chemotherapy, which frequently leads to resistance and severe toxicity (44, 45). Our findings suggest that targeting the Wnt-ferroptosis axis—combining pathway inhibition with regulated cell death induction—represents a novel approach to overcome chemoresistance. Promising combination strategies include LF3 with conventional chemotherapeutics (doxorubicin, cisplatin), with ferroptosis inducers (RSL3, erastin), or with immune checkpoint inhibitors given emerging evidence linking ferroptosis to immunogenic cell death (46).

Several limitations should be acknowledged. First, only two cell lines were evaluated; future studies should incorporate patient-derived xenograft (PDX) models to better capture disease heterogeneity. Second, although we demonstrate that LF3 simultaneously inhibits Wnt signaling and induces iron death, the exact molecular cascade by which TCF4 inhibition leads to HO-1 and ACSL4 upregulation remains to be fully elucidated. Whether TCF4 represses these genes by binding directly to promoter regions or acts through intermediate transcriptional networks (e.g., Nrf2/ARE or Wnt-dependent lipid metabolism reprogramming) requires validation by chromatin immunoprecipitation sequencing (ChIP-seq), promoter reporter assays, and comprehensive transcriptomic approaches. At present, the above mechanistic studies are being carried out in our laboratory and will be the focus of subsequent study reports. Third, orthotopic models would more faithfully recapitulate the bone microenvironment. Fourth, long-term safety, resistance development, and pharmacokinetic optimization require comprehensive evaluation prior to clinical translation. The concentration of 40 μM LF3 used for *in vitro* experiments was based on dose-response curves and was able to produce consistent, statistically robust biological effects across multiple assays. However, whether equivalent concentrations can be achieved in osteosarcoma tumor tissue at clinically tolerable systemic doses has not been validated by formal pharmacokinetic studies. Future studies should include plasma concentration-time curve analysis, tumor tissue distribution analysis, and determination of tissue-to-plasma concentration ratios to clarify the relationship between *in vitro* effective concentrations and *in vivo* dosing regimens, which are important prerequisites for clinical translation of LF3.

In conclusion, this study establishes LF3 as a dual-targeting therapeutic agent that suppresses osteosarcoma through coordinated Wnt pathway inhibition and *HO-1/ACSL4*-mediated ferroptosis induction. These findings advance understanding of Wnt-ferroptosis crosstalk and provide a rational foundation for developing novel combination therapies for chemoresistant osteosarcoma.

## Data Availability

The datasets presented in this study can be found in online repositories. The names of the repository/repositories and accession number(s) can be found in the article/**Supplementary Material**.
