# Supplementary material for: LF3 exerts anti-osteosarcoma effects through Wnt signaling inhibition-coupled ferroptosis induction via HO-1/ACSL4 axis
Source: Front Oncol. 2026 May 12;16:1832247. doi: 10.3389/fonc.2026.1832247 (PMC13201497; doi:10.3389/fonc.2026.1832247)
Supplement: Supplementary file 1 [file Table1.docx]

**Supplementary Table**

**Supplementary Table 1. The forward and reverse primers used for qPCR.**

| **Gene** | **Primer sequence**  **(forward/reverse/****5'-3')** | **Gene** | **Primer sequence**  **(forward/reverse/5'-3')** |
| --- | --- | --- | --- |
| AXIN2 | CCCTGCCCCACAAAGAAATG | BMP4 | GTGGGCTGGAATGACTGGAT |
|  | ACCCGAACATCAAAATGAAGGTG |  | GGTTGGTTGAGTTGAGGTGGT |
| SAMD6 | TCTGTTGTTCCTTCCTGCCCA | LEF1 | TAGTGGCTTCTCCGCCCTTG |
|  | GTATTCTTTTCCCCACCGCCA |  | CAACAGAGTGGGTTTGGCTATTACA |
| TFR1 | TCAGCAGAGACCACCGAAGACT | FTH1 | TGAAGCTGCAGAACCAACGAGG |
|  | GACCACACTTGCCCGCTATGTA |  | GCACACTCCATTGCATTCAGCC |
| HO1 | CCAGGCAGAGAATGCTGAGTTC | GAPDH | ACATCAAGAAGGTGGTGAAGCA |
|  | AAGACTGGGCTCTCCTTGTTGC |  | CGTCAAAGGTGGAGGAGTGG |
| DPP4 | AATCACATGGACGGGGAAAGAAG | TYRO3 | CGGATGTTGGGCAAAGGAGAG |
|  | AGACCACCACAGAGCAGAGTAG |  | TCGCTTGAGGCAATGATGTCAG |

**Supplementary Table 2. Brand and dilution ratio of antibodies used in Western blot.**

| Antibody | Manufacturer and Catalog | Dilution Ratios |
| --- | --- | --- |
| Anti-Transferrin Receptor (CD71) Antibody (TFR1) | HUABIO / ET1702-06 | 1：1000 |
| Anti-Heme Oxygenasel Antibody(HO-1) | HUABIO / ET1604-45 | 1：1000 |
| Anti-Ferritin Heavy Chain Antibody(FTH1) | HUABIO / ET1705-55 | 1：500 |
| MBOA5 Rabbit pAb  (LPCAT3) | Immunoway Biotechnology/YT8099 | 1：1000 |
| [ARA70 Rabbit pAb](https://www.immunoway.com/products/primary-antibodies/YT0302-ARA70-Rabbit-pAb.html)(NCOA4) | Immunoway Biotechnology/YT0302 | 1：1000 |
| Anti-TCF7L2/TCF4 Antibody(TCF4) | HUABIO / ET1610-73 | 1：500 |
| β-Catenin Antibody(β-Catenin ) | Cell Signaling Technology / #9562 | 1：5000 |
| Cleaved-Caspase 1 (Asp296), p20 Antibody  (caspase-1 p20) | Affinity Biosciences/AF4005 | 1：500 |
| LC3B Rabbit mAb (LC3A/B) | ABclonal/ A15591 | 1：500 |
| Anti-GAPDH Antibody(GAPDH) | HUABIO / ET1601-4 | 1：5000 |
| ACSL4 (PT0448R) Rabbit mAb  (ACSL4) | Immunoway Biotechnology/YM8287 | 1:1000 |
| HRP Conjugated Goat anti-Rabbit IgG Antibody | HUABIO / HA1001 | 1:50000 |

**Supplementary Table 3. Brand and dilution ratio of antibodies used in immunohistochemistry.**

| Antibody | Manufacturer and catalog | Dilution ratios |
| --- | --- | --- |
| 4-HNE | MedChemExpress,Monmouth Junction, NJ, USA, HY-P81208 | 1: 100 |
| Ki67 | Immunoway Biotechnology, China YM8189 | 1: 100 |
| Goat Anti-Rabbit IgG H&L | HUABIO, China, No. HA1001 | 1: 50000 |
| HO-1 | HUABIO, China, ET1604-45 | 1:50 |
| ACSL4 | Immunoway Biotechnology, China, YM8287 | 1:200 |
